# Predator recognition and differential behavioural responses of adult wood warblers *Phylloscopus sibilatrix*

**DOI:** 10.1007/s10211-017-0275-2

**Published:** 2017-09-05

**Authors:** Marta Maziarz, Charlotte Piggott, Malcolm Burgess

**Affiliations:** 1 PiedFly.Net, Yarner Wood, Bovey Tracey, Devon TQ13 9LJ UK; 2 Centre for Research in Animal Behaviour, College of Life & Environmental Sciences, University of Exeter, Exeter, EX4 4QG UK; 3 RSPB Centre for Conservation Science, The Lodge, Sandy, Bedfordshire SG19 2DL UK

**Keywords:** Nest defence, Anti-predator behaviour, Provisioning, Call rate, Nest attendance

## Abstract

Birds often engage in nest defence against predators to improve breeding success, but defence efficiency requires the capability to assess the threat level posed by potential predators. For species with low breeding-site tenacity, which may encounter varying occurrence and density of predators in different areas, threat recognition could be compromised due to naivety, and so predator recognition may focus on broad key features to diminish the risk of misidentification. We experimentally tested this hypothesis by recording behavioural reactions of the nomadic wood warbler *Phylloscopus sibilatrix* to objects reflecting various levels of threat: least weasel and Eurasian jay taxidermy mounts, an inanimate object and an empty display mount. To assess actual nest predators, we used remote cameras to record predation events at wood warbler nests. As in other studies in Western Europe, Eurasian jay was found to be the main nest predator, with occasional predation by least weasel. The reaction of adult warblers to the models was generally to remain silent and on nests during the incubation stage presumably due to the need to maintain efficient nest camouflage and concealment. During the nestling stage, behavioural responses of adult warblers, calling and suspended feeding of young, showed the strongest effects from the jay taxidermy mount, moderate to the weasel and weakest to the inanimate object and empty mount. As the reaction of wood warblers reflected the degree of genuine threat posed by the predators depicted by the models, we conclude that predator recognition may be present in this species.

## Introduction

Parental investment in active nest defence to protect eggs and nestlings against predators can increase the reproductive success of birds (e.g. Edmunds 1974; Montgomerie and Weatherhead 1988). For nest defence to be effective, parent birds need to distinguish between different levels of threat posed by potential predators and harmless situations and employ an appropriate response relative to the level of danger to prevent the nest being detected and predated (Curio 1993; Caro 2005). Recognition of predators that pose the greatest threat would be advantageous for optimal nest defence. If all predators pose a similar threat, the response of parent birds would be consistent, and so recognition at the most general level would be sufficient (McLean and Rhodes 1991).

Predator recognition by birds can be innate (e.g. Tinbergen 1951; Curio 1975) and/or acquired over time through learning (McLean and Rhodes 1991; Maloney and McLean 1995; Kullberg and Lind 2002; Bogrand et al. 2017). For species with low breeding-site tenacity, which may encounter varying predator species in different areas, recognition of threat based on previous experience could be costly as the risk of misidentification would be elevated during encounters with novel predators. It could be expected that in such cases, predator recognition should be mainly innate and involve recognition of the broad key features of a predator, but it remains unclear whether this is the case.

Although the parental reaction to potential nest predators may differ with an ability to detect specific threats, vigilance may also vary due to other factors (Montgomerie and Weatherhead 1988; McLean and Rhodes 1991; Bures and Pavel 2003; Caro 2005). For example, nest defence by adult birds can be more vigorous against predators when parental investment and the risk of nest loss are both high (e.g. Greig-Smith 1980; Redondo 1989). Additionally, there may be a trade-off between active nest defence and the need to remain quiet to avoid revealing the location of well-concealed or camouflaged nests (e.g. Edmunds 1974; Burhans 2000; Bures and Pavel 2003). Active nest defence by parents can thus be considered a dynamic decision-making process that depends on birds’ assessment of the costs and advantages of a given situation (Montgomerie and Weatherhead 1988; Kleindorfer et al. 2005).

The extent to which passerines with low site fidelity can discriminate between nest predators and differentially react to them at different stages of the nesting cycle is little explored. In this study, we examined behavioural responses of the wood warbler *Phylloscopus sibilatrix*, a migratory passerine showing low breeding-site fidelity with adult return rates to breeding-sites varying from 0% in Eastern Europe to 28% in Britain (Wesołowski et al. 2009). Wood warblers show high inter-annual fluctuations in local breeding numbers which is not linked to variation in local production of young or survival (RSPB, unpublished data), and therefore must involve large-scale irruptions in some years and emigration in others. Wood warblers are ground-nesting, building domed nests consisting of grasses, leaves and moss that are easily accessible to many potential predators, and predation is the main cause of nest failure (e.g. Wesołowski and Maziarz 2009; Mallord et al. 2012; Grendelmeier et al. 2015). Predation of adult females at the nest also occurs (e.g. Wesołowski 1985; RSPB, unpublished data).

As a result of their low site fidelity, wood warblers probably come into contact with a great diversity of predators while breeding in different areas over successive years, and require vigilance to a wide spectrum of potential threats to nests (Mallord et al. 2012; Grendelmeier et al. 2015). Thus, the threat these predators pose presumably also differs in importance to nesting wood warblers across their breeding range.

Current knowledge of wood warbler reaction to the presence of predators near nests includes only general observations of responses to a range of potential threats including great spotted woodpecker *Dendrocopos major*, Eurasian jay *Garrulus glandarius*, cuckoo *Cuculus canorus*, adders *Vipera* spp., squirrels *Sciurus* spp., small rodents such as mice *Apodemus* spp., and larger animals including deer, dogs and man; all of them elicit alarm behaviour in wood warblers (e.g. Treuenfels 1937, 1940; Aschenbrenner 1966; Fouarge 1968; Hammer 1975). Similar alarm reactions to all potential predators would suggest a limited ability to discriminate between different levels of risk, but this assumption has not previously been tested.

Furthermore, as wood warbler nests are usually cryptic and well concealed (Wesołowski 1985; Cramp 1992), adults might modify their own response to nest predators in ways that maximise the efficacy of nest camouflage and concealment. It could be presumed that birds would show little vigilance during the egg-laying and incubation stages, when parental activity at the nest is low and nest camouflage or concealment play a major role in nest defence (Grendelmeier et al. 2015). By contrast, alarm responses may increase during the nestling stage when parental investment and the risk of nest loss are greatest (e.g. Wesołowski 1985; Wesołowski and Maziarz 2009; Mallord et al. 2012; Grendelmeier et al. 2015) and nest camouflage and concealment become least effective (Grendelmeier et al. 2015).

Here, we experimentally test the hypotheses that adult wood warblers are unable to discriminate between different levels of danger close to their nests and show a consistent behavioural response to all potential threats, as previous observations on wood warblers would suggest. If however they are able to discriminate between threats, we expect the response of adults to be absent or weak to the presence of harmless innate objects, and increase to the presence of predators, and be strongest to the predators which pose the greatest actual danger. Additionally, we presume that wood warblers would adjust their reactions to potential predation risk depending on the nest stage, showing stronger response in the nestling period than in the incubation period when parental investment in nests is lower.

## Methods

### Study site and nest monitoring

Experiments were conducted in 2012 at Yarner Wood, part of East Dartmoor National Nature Reserve situated on Dartmoor, Devon, UK (50° 36′ N, 3° 43′ W). Yarner Wood is a 150-ha upland woodland dominated by a sessile oak *Quercus petrea* canopy, a rowan *Sorbus aucuparia* and European holly *Ilex aquifolium* understory and a dense European blueberry *Vaccinium myrtillus* field layer, with elevation ranging from 150 to 300 m.

Wood warbler nests were located by following adults back to nests. Adults make regular calls throughout the nesting cycle as they descend from trees to nests on the ground, especially when nest building and provisioning young. Located nests were then visited every 3–4 days and monitored until young fledged or the nest failed. First egg-laying dates were either determined from visits during egg laying assuming that one egg is laid per day, or were back-calculated after hatching assuming a 13-days incubation period and incubation starting the day the last egg was laid (Cramp 1992). First-egg dates were used to calculate the initiation of the incubation stage.

### Identity of nest predators

To identify which predator species locally pose the greatest threat to nesting wood warblers, we used remote cameras deployed at nests at Yarner Wood and in 12 other deciduous woodlands spread across the Dartmoor area. Nest monitoring with nest cameras was conducted in 2012 and 2013. Nest cameras were purpose-built; they had high responsiveness, worked 24 h per day and typically captured several images of predation events. The camera and software used were identical to that described in Bolton et al. (2007). Cameras were deployed 0.5–1.5 m from nests and were powered by 12-V batteries situated with recording units several meters further away as described in Mallord et al. (2012). All equipment was covered and camouflaged with leaf litter. Nest cameras were initially deployed randomly at nests at the egg stage and subsequently were redeployed on nests at any stage of development to maximise the number of nests monitored by cameras and the chances of recording predation. Only nests that had predator identity determined by nest cameras were included in the analysis. We included all cases of predator attack on nests regardless of whether any eggs or young survived. This included seven attacks on nests with young ≥ 9 days old, which would be capable of escaping the nest and surviving a predator attack (Wesołowski and Maziarz 2009).

### Predator simulation experiment

To experimentally test wood warbler responses to different predators, we used taxidermy mounts and an inanimate object which were presented at two stages of the nesting cycle, once during the incubation (May 23rd to June 1st; median date = May 29th) and once at the nestling stage (June 2nd to June 26th; median date = June 6th). We used mounts of Eurasian jay and least weasel *Mustela nivalis* (hereafter jay and weasel), a metal cup and an empty display mount (the presentation box but with no mount or object) for comparison. Both jay and weasel are widespread predator species found across Eurasia (Hagemeijer and Blair 1997). The jay is the most frequent predator of wood warbler nests at both egg and chick stages (Mallord et al. 2012; Grendelmeier et al. 2015). The weasel has not been recorded in the literature as a predator of wood warbler nests, but is a common predator of other bird species nests (e.g. Dunn 1977; Weidinger 2009). The mounts of these predator species were selected to reflect different levels of threat to nesting wood warblers.

We used a purpose-made box (24 cm high × 18 cm wide × 53 cm long) to present each mount so that it could be remotely operated 15–25 m away from nests to avoid an observer influence on bird behaviour. The presentation box was located on the ground, 2–3 m from the nest entrance, i.e. within the threshold distance where the presence of predators should elicit a reaction (based on personal observations from installing nest cameras in previous years). The box was in position 20 min before the experiment began to allow parents to resume natural behaviours (incubation or feeding young). The mount or object was located at the top of the box and was not visible until the observer remotely opened the presentation box from several meters away with a pull cord. Experiments always began with the presentation of an empty mount, followed in a random order by jay, weasel and mug.

In each experiment, the mount was exposed for 3 min, but vocal and behavioural responses of adults were recorded from 20 min before the mount exposure until 20 min after the mount had been concealed from view. This was repeated for each mount or object (jay, weasel, mug and empty mount) so that each nest was subjected to all four presentations, with a minimum interval of c. 10 min between treatments to replace the mount in the box. In consequence, breaks between the model presentations lasted for a minimum of 50 min, thereby avoiding birds potentially becoming habituated to the presentation box (Hinde 1954). Efforts were made to conduct all four experiments at individual nests on the same day, but at three nests, the experiments had to be split over 2 days due to heavy rain.

Continuous audio recording of vocalisations was made in MP3 file format using a Marantz PMD671 model solid-state recorder and directional microphone placed 10–15 m from the nest and camouflaged with leaf litter. Parental attendance and latency to leave and return to nests was recorded by the observer from approximately 20 m from the nest that ensured observer presence did not influence bird behaviour. The experiments were initiated only after ascertaining that the female was on the nest during incubation stage experiments or was feeding and not brooding young at nestling stage experiments.

### Data analysis

The behavioural responses of parents to model predators was analysed based on the sound recordings and direct observations collected during the period of mount presentation and the period immediately following it. This ensured that the measured reaction of adults related solely to mount presence and no other disturbances unrelated to the experiment, such as the presence of natural predators. We used the remaining parts of the recordings and direct observations as an additional source of information on the wood warbler reaction to the presence of live predators (‘natural’ disturbances).

With the appearance of predators, wood warblers often suspend activity at the nest and begin to call (Cramp 1992), so to measure the level of parent alarm during the experiments we recorded the following:

- at incubation stage:

- total number of calls produced by a female that left the nest during or after model presentation. In cases when a female was silent and remained inside or outside of the nest, the number of calls was recorded as 0.
- time latency (seconds) of the female leaving the nest after the mount was shown, measured until model was hidden.
- time latency (seconds) of the female leaving the nest after the mount was concealed.

b) at nestling stage:

- the number of calls produced by a parent; calls by two parents were often given at different distances from the microphone so they could be distinguished; thus, the number of calls produced by one parent should not be overestimated. During model presentation, the number of calls corresponded to those given between the mount display and the first parental visit to the nest if this occurred before the model was concealed. In such cases, the number of calls in the period following model concealment was taken as 0. When the first feeding visit occurred after the model was concealed, the number of calls that were produced by a parent before the first visit to the nest was divided between the two presentation stages (before and after model concealment).
- feeding time latency (seconds) from mount exposure until the first adult visit to the nest.
- the number of parent (feeding) visits made during the 3-min presentation of the mount and the following 20 min, expressed as the number of feeding visits per minute of each observation stage calculated separately.

Responses in call rate, feeding visits and latency to first feeding visit after model presentation were analysed using pairwise Wilcoxon matched pair sign tests. Cohen’s *d* was calculated to determine effect size (Cohen 1992), with *d* values < 0.2 being considered negligible effects, between 0.2 and 0.49 small effects, 0.5–0.79 medium and > 0.8 large effects. Due to nest losses, experiments at some nests could not be repeated during the nestling stage. To increase the sample size in the nestling stage, some later found nests were included. The sample sizes during the incubation and the nestling stages therefore contained different sets of nests. Statistical comparisons of responses across the two nesting stages could not be performed as this would require excluding later found nests and therefore insufficient sample sizes. All statistical analyses were performed in R version 3.3.2 (R Development Core Team 2016).

## Results

### Identity of nest predators

Nest cameras monitored 66 nests, of which 34 were predated. Predation mostly occurred at the nestling stage (31 nests) and rarely at the egg stage (3 nests). The most frequently identified predators were common buzzard *Buteo buteo* (8), jay (7) and Eurasian sparrowhawk *Accipiter nisus* (4), these collectively being responsible for 56% of recorded predation events (Table 1). Mammals predated ten nests and were represented by six species, including two instances of weasel (Table 1), one resulting in predation of the female as well as the nestlings.

**Table 1:** Number of wood warbler nests predated or partially predated by different predators detected by nest cameras across Dartmoor, Southwest England. Forty-one nests were monitored with cameras in 2012 and 25 in 2013

Predator species	2012	2013	Total
Common buzzard *Buteo buteo*	4	4	8
Eurasian jay *Garrulus glandarius*	6	1	7
Eurasian sparrowhawk *Accipiter nisus*	4	0	4
European badger *Meles meles*	0	2	2
Grey squirrel *Sciurus carolinensis*	2	0	2
Least weasel *Mustela nivalis*	1	1	2
Mouse or vole	1	1	2
Brown rat *Rattus norvegicus*	1	0	1
Red fox *Vulpes vulpes*	0	1	1
Unknown	3	2	5
Total	22	12	34

### Adult response during the incubation stage

Experiments were conducted at nine nests, from 3 to 11 days after incubation was initiated (median 7 days). At incubation stage, females showed little reaction to all model presentations, remaining on the nest when mounts of jay or weasel were revealed and leaving one nest when presented with an empty mount and at two when presented with the mug (latency to leave nests occurring 17–19 s after models were revealed). One female left the nest in the 20 min period before an empty mount presentation, returning within 5 min of the mount being hidden. The mean number of calls produced by a parent at the nest was low during presentations, ranging from 0 during jay and weasel displays to 1.2 (SD = 3.3) calls during the mug and 2.4 (SD = 7.3) during the empty mount exposure. The effect sizes of call rates between matched pairs were small to medium, (Cohen’s *d* < 0.53 in all pairings) and all Wilcoxon sign tests were non-significant (*P* > 0.05).

After mount concealment, the females that remained on nests during the jay and weasel presentations left nests a median of 5 min after jay mount concealment and 8 min after weasel mount concealment (Table 2). Conversely, four of nine females did not react after the mug or empty mount was concealed and so continued to incubate for the entire experiment (Table 2). Call rates in the 20 min after mount concealment varied between mounts (means: jay 28.6, weasel 38.6, mug 47.8 and empty mount 27.6 calls), but effect sizes of call rates between matched pairs were all small (Cohen’s *d* < 0.38 in all pairs) and all Wilcoxon sign tests were non-significant (*P* > 0.05).

**Table 2:** The time latency (seconds) between mount presentations being concealed and females leaving the nest, for each mount presented at the incubation stage at nine nests tested

Mount presented	Females leaving nest (*n*)	Latency (s) to leave the nest	
		Median	range
Empty mount	3	349	277–893
Mug	3	830	0–869
Weasel	5	480	151–1097
Jay	7	294	0–893

### Adult response during the nestling stage

**Fig. 1:**
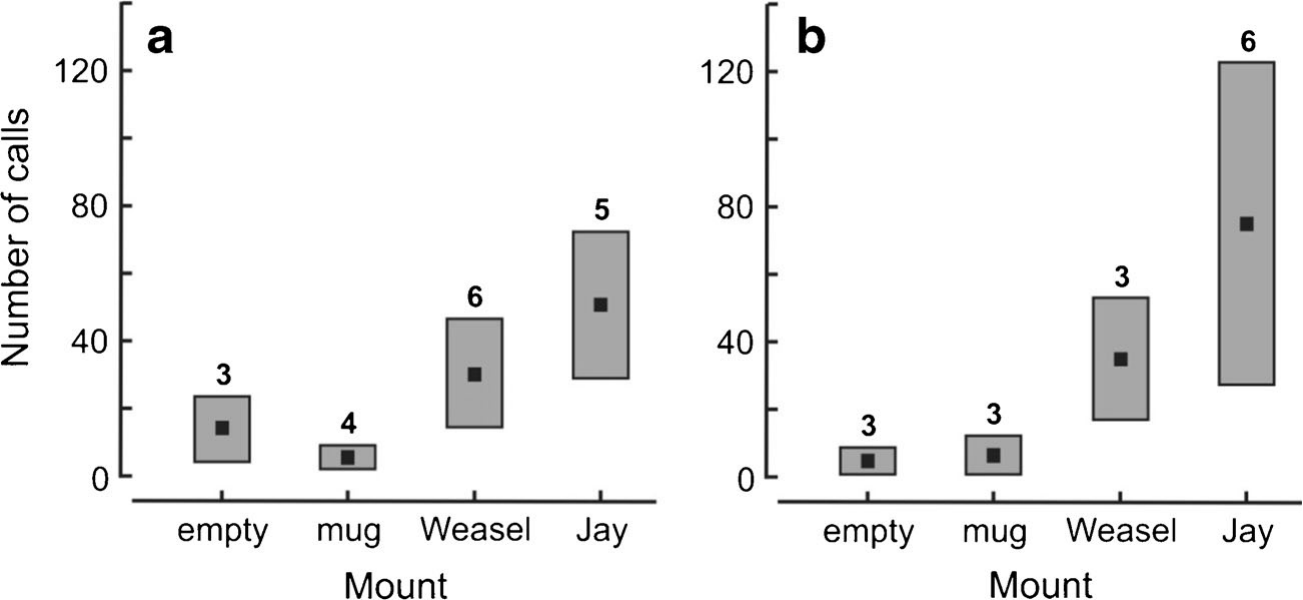
The number of calls given by wood warbler parents at the nest: **a** during and **b** after presentation of an empty mount, mug and taxidermy mount of a least weasel *Mustela nivalis* and Eurasian jay *Garrulus glandarius* during the nestling stage*.* Shown are means (black squares) and standard errors (rectangles). In each treatment, the reaction of parent birds was tested at eight nests. The number of nests at which calling occurred is given above the bars

Nestling stage experiments were conducted at eight nests, when chicks were 3–9 days old (median 4 days). At this stage, the presentations of jay or weasel elicited a calling reaction by adults at most nests (respectively at five and six nests, Fig. 1a). A single parent returned to the nest at the three of the eight nests, and at two of these nests began to call intensively immediately after first visiting and continued calling until after the mount was concealed. Calling was less frequent during the presentations of the mug and empty mount (Fig. 1a). The effect size of call rates between matched pairs during displays showed a large effect size to the jay compared to the mug (Cohen’s *d* = 1.066, Wilcoxon sign test: *Z* = 1.914, *P* = 0.056), and medium effects of jay with the empty mount (Cohen’s *d* = 0.776, Wilcoxon sign test: *Z* = 1.451, *P* = 0.147) and between the weasel and mug (Cohen’s *d* = 0.793, Wilcoxon sign test: *Z* = 1.845, *P* = 0.065).

After mounts were hidden, the alarm response of adults to the jay mount was still detected, with calling maintained at six nests following jay concealment and at only three nests following all other mounts combined (Fig. 1b). The mean number of calls recorded after the jay display was much higher than all the other mounts (Fig. 1b). The number of calls recorded following weasel display was less than half compared to the jay but still higher compared to the mug and empty mount. The effect size of call rates between matched pairs after displays showed a large effect size of the weasel compared to the empty mount (Cohen’s *d* = 0.833, Wilcoxon sign test: *Z* = 1.213, *P* = 0.225) and medium effects of jay with the mug (Cohen’s *d* = 0.714, Wilcoxon sign test: *Z* = 2.339, *P* = 0.019), jay and empty mount (Cohen’s *d* = 0.739, Wilcoxon sign test: *Z* = 1.596, *P* = 0.111) and between the weasel and mug (Cohen’s *d* = 0.755, Wilcoxon sign test: *Z* = 1.213, *P* = 0.225).

**Fig. 2:**
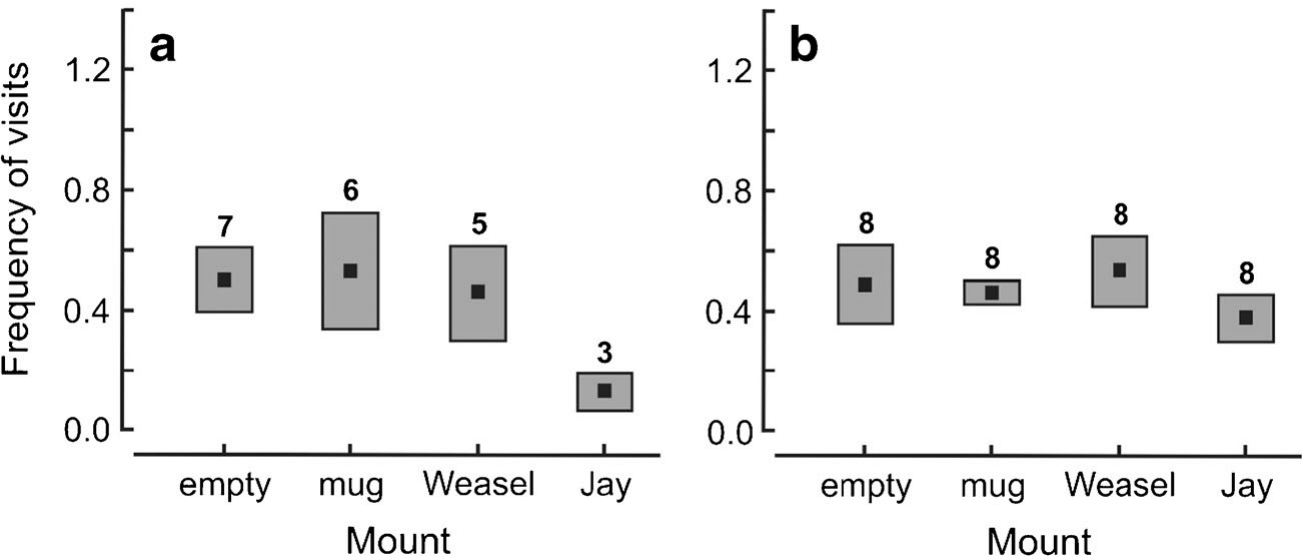
The frequency of feeding visits (number per minute) by wood warbler parents to nests containing nestlings: **a** during the 3 min of presentation and **b** during the 20 min after an empty mount, mug and a taxidermy model of least weasel *Mustela nivalis* and Eurasian jay *Garrulus glandarius* were concealed*.* Shown are means (black squares) and standard errors (bars). In each treatment, the reaction of parent birds was tested at eight nests. The number of nests at which a feeding visit was recorded is given above the bars

The number of feeding visits to nests was unaffected during the empty mount, mug or weasel displays, but most parents suspended feeding of young in reaction to the presence of the jay (Fig. 2a). A single parent returned to the nest at the three of the eight nests, but only fed once before calling until after the mount was concealed, presumably not noticing the threat until visiting the nest. The effect size of feeding rates between matched pairs during displays showed a large effect size to the jay compared to the mug (Cohen’s *d* = 1.052, Wilcoxon sign test: *Z* = 1.605, *P* = 0.109), the empty mount (Cohen’s *d* = 1.500, Wilcoxon sign test: *Z* = 2.369, *P* = 0.018) and weasel (Cohen’s *d* = 1.009, Wilcoxon sign test: *Z* = 1.783, *P* = 0.075) with all other effect sizes being negligible.

The number of feeding visits during the 20 min following mount concealment was higher for all mounts compared to during displays, and was lowest following the jay mount and highest following the weasel (Fig. 2b). Call rates were similar across the different mounts and effect sizes were negligible or small for all pairs, the greatest effect being between jay and weasel (Cohen’s *d* = 0.506, Wilcoxon sign test: *Z* = 1.845, *P* = 0.065).

**Fig. 3:**
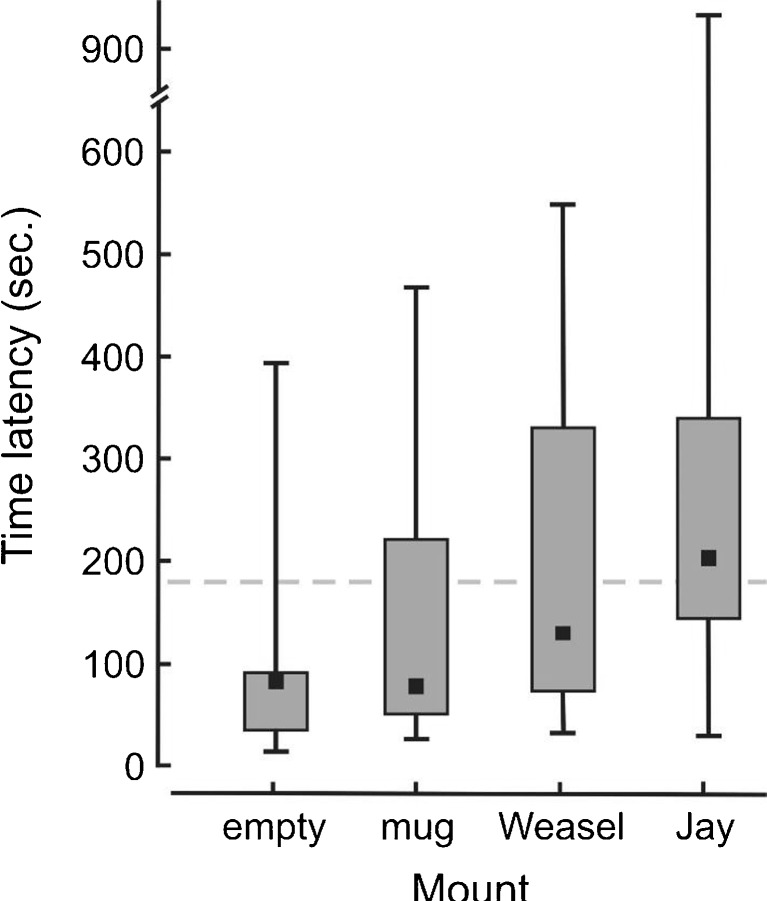
Time latency of the first feeding visit by a wood warbler parent to the nest following presentation of an empty mount, mug and a taxidermy mount of least weasel *Mustela nivalis* and Eurasian jay *Garrulus glandarius*. Shown are medians (black squares), 25th–75th percentiles (bars) and min-max values (whiskers). Mounts were presented for 180 s, and the dotted line indicates when mounts were concealed

The latency between mount presentation and the first subsequent feeding visit to the nest was most delayed for the jay mount (Fig. 3). In reaction to the jay, parents resumed feeding young typically just after the mount was concealed, whereas for the remaining mounts, first visits typically occurred during mount display. The effect sizes of latency to feed young between matched pairs showed a large effect between the jay and empty mount (Cohen’s *d* = 0.831, Wilcoxon sign test: *Z* = 1.960, *P* = 0.05) and medium effects between jay and mug (Cohen’s *d* = 0.618, Wilcoxon sign test: *Z* = 0.911, *P* = 0.362), and between weasel and the empty mount (Cohen’s *d* = 0.640, Wilcoxon sign test: *Z* = 1.540, *P* = 0.124).

## Discussion

The differences observed in the response of wood warblers to the mounts and objects presented at nests varied between the two nest stages. During the incubation stage, when females spend most time on the nest and the male rarely visits (Cramp 1992), the reaction of females was negligible when jay or weasel mounts were presented with no females calling or leaving nests. Occasional vocal behaviours were recorded when an empty mount or a mug was shown, and most females left nests following model concealment. At the nestling stage, presentation of the taxidermy jay and weasel often elicited calling by parents, but the behavioural response of adults (including suspension of feeding young) was particularly strong to the jay mount. In contrast, parents reacted less to the presence of the mug or empty mount and continued feeding their young silently during these presentations.

Reactions of wood warblers to actual live predators in the vicinity of nests, observed in this and previous studies (Treuenfels 1937, 1940; Fouarge 1968; Hammer 1975), were similar to those recorded during the experiments with the jay and weasel mounts. In one of our experiments during the incubation stage, a real jay appeared after a mug was concealed following presentation. In this case, the female wood warbler remained on the nest and did not call. Such behaviour of females has also been observed elsewhere in situations when potential predators passed a nest, with incubating female wood warblers usually remaining on the nest and only taking flight at the last moment to perform a distraction display (Cramp 1992). At the nestling stage, a live jay was seen twice in the vicinity of a nest, once before and once sometime after the presentation of the mug when the birds had resumed normal activity. On each occasion, the parent wood warblers called intensively and suspended feeding visits to the nest.

The increased response of wood warblers to mounts with the advancement of breeding could be partially explained by higher parental investment and/or risk of predation, both exerting greater pressure on parents to actively defend their nests at the nestling stage (e.g. Greig-Smith 1980; Montgomerie and Weatherhead 1988; McLean and Rhodes 1991; Randler 2013). In our study, as in other wood warbler studies (Wesołowski 1985; Wesołowski and Maziarz 2009; Mallord et al. 2012; Grendelmeier et al. 2015), predation rates were higher at the nestling compared to egg stage. At the nestling stage, when parents frequently visit the nest to feed young, the efficacy of nest and adult camouflage decreases (Skutch 1949; Edmunds 1974; Caro 2005). Thus, in order to prevent predation, parents may enhance nest concealment at the critical moment when a predator approaches the nest, for example by quietening young and suspending feeding visits (Halupka 1998). Calling loudly could warn young and mate of potential danger, and distract predators from finding the nest (e.g. Greig-Smith 1980; Montgomerie and Weatherhead 1988; Halupka 1998; Gill and Sealy 2003; Platzen and Magrath 2004; Randler 2013). Similar behaviour of adults is observed in many other species relying on nest concealment (e.g. Halupka 1998; Burhans 2000; Bures and Pavel 2003), suggesting that use of calls and suspension of feeding by parents may be a general anti-predator method used by birds at the nestling stage.

Contrary to our expectations, reactions of adults to the mounts suggested that parent wood warblers were able to discriminate between the different levels of threat represented by the four objects. These differences in behaviour were apparent despite some limitations of the study. The small sample size during the incubation and nestling stages, or individual variation in adult reaction (personality), might mask patterns in behaviour and decrease the detectable differences. A further limitation of the experiment was the static mounts, thereby not reflecting natural predator movement, and so the recorded responses could reflect a reduced level of stimulus or agitation compared to an actively moving predator near the nest (Curio 1993).

The patterns of wood warbler reactions matched the real level of threat. In the Dartmoor area, wood warbler nests were most frequently predated by avian predators, mainly jay and common buzzard, while weasel predation was recorded twice. Jay is one of the main wood warbler nest predators across Western Europe, being responsible for 60% of nest predation in Wales (UK) and 29% in Switzerland (Mallord et al. 2012; Grendelmeier et al. 2015), although not recorded in a Central European study (Białowieża Forest, Poland; Maziarz et al. in prep.). In contrast, weasel was not previously reported as a wood warbler nest predator. Hence, a generally stronger response of parents to the jay, a more frequent predator of wood warbler nests than weasel, may reflect the reaction of adults to the degree of actual risk posed by these species. Also, a weaker response to the mug or empty mount compared to the taxidermy mounts suggests that wood warblers were able to assess if a potential threat was present near the nest. Our findings are in contrast to many previous studies that show consistent reactions of birds to various potential predators (e.g. Treuenfels 1937, 1940; Aschenbrenner 1966; Fouarge 1968; Hammer 1975), suggesting often limited abilities of birds generally to discriminate between different levels of threat.

Although jay and weasel are both abundant and widespread species across Europe and common predators of bird nests (e.g. Dunn 1977; Weidinger 2009), when defending nests, wood warblers probably come into direct contact with jays more often during their life compared with weasels. Consequently, recognition of jays as nest predators might be based on individuals previous experience to some extent and so may involve recognising species-specific features, while recognition of weasels is more likely to be innate and follow more general features (e.g. McLean and Rhodes 1991; Bogrand et al. 2017). Therefore, it is possible that wood warblers, which show low site tenacity (Wesołowski et al. 2009), use two techniques in assessing predation risk: species-specific recognition of more threatening predators encountered commonly and the recognition of general characteristics such as movement (Treuenfels 1940; Curio 1993) of more ‘unpredictable’ predators encountered infrequently.

Parental vocalisations of many bird species differ in response to risks from avian or mammalian predators (e.g. Gottfried et al. 1985; Gill and Sealy 2003; Randler 2013). This does not seem the case in wood warblers which have a simple and uniform vocalisation (Cramp 1992). Thus, passing information about the urgency of the threat rather than about the type of the predator appears to be the primary function of the alarm vocalisations in this species.

In summary, this study suggests that the wood warbler, like other bird species (e.g. Gottfried et al. 1985; Maloney and McLean 1995; Burhans 2000; Bures and Pavel 2003; Gill and Sealy 2003; Bogrand et al. 2017), are probably capable of distinguishing between different levels of threat. We found wood warblers tended to react most strongly to predators which posed the greatest actual threat, and at the nestling stage when threats are greater and parental investment has been highest.
